# Optimizing renewable-based energy supply options for power generation in Ethiopia

**DOI:** 10.1371/journal.pone.0262595

**Published:** 2022-01-14

**Authors:** Megersa Tesfaye Boke, Semu Ayalew Moges, Zeleke Agide Dejen

**Affiliations:** 1 Addis Ababa University, Ethiopian Institute of Water Resources (EIWR), Addis Ababa, Ethiopia; 2 School of Civil and Environmental Engineering, Connecticut University, Storrs, CT, United States of America; Universiti Sains Malaysia (USM), MALAYSIA

## Abstract

Ethiopia unveiled homegrown economic reform agenda aimed to achieve a lower-middle status by 2030 and sustain its economic growth to achieve medium-middle and higher-middle status by 2040 and 2050 respectively. In this study, we evaluated the optimal renewable energy mix for power generation and associated investment costs for the country to progressively achieve upper-middle-income countries by 2050. Two economic scenarios: business as usual and Ethiopia’s homegrown reform agenda scenario were considered. The study used an Open Source energy Modeling System. The model results suggest: if projected power demand increases as anticipated in the homegrown reform agenda scenario, Ethiopia requires to expand the installed power capacity to 31.22GW, 112.45GW and 334.27GW to cover the current unmet and achieve lower, medium and higher middle-income status by 2030, 2040 and 2050 respectively. The Ethiopian energy mix continues to be dominated by hydropower and starts gradually shifting to solar and wind energy development towards 2050 as a least-cost energy supply option. The results also indicate Ethiopia needs to invest about 70 billion US$ on power plant investments for the period 2021–2030 to achieve the lower-middle-income electricity per capita consumption target by 2030 and staggering cumulative investment in the order of 750 billion US$ from 2031 to 2050 inclusive to achieve upper-middle-income electricity consumption rates by 2050. Ethiopia has enough renewable energy potential to achieve its economic target. Investment and financial sourcing remain a priority challenge. The findings could be useful in supporting decision-making concerning socio-economic development and investment pathways in the country.

## 1. Introduction

Energy is an important element in accomplishing interrelated socio-economic development and is a backbone of a modern economy [1–4]. A quantitative relationship between energy use and economic growth has been well documented for countries in different phases of development [5–9]. Given growing demand due to socio-economic transformations, energy resources become relatively scarce. This in turn has an impact on the future availability of resources for long-term economic development. The pursuit of optimal supply options can thus be viewed as the backbone of any country’s long-term economic and social prosperity.

Ethiopia is located on the horn of Africa, in the east of the continent, located between the Equator and the Tropic of Cancer, between 3^0^ and 15^0^ N latitude and 33^0^ and 48^0^ E longitude and is one of the few countries in the world where the electricity grid is nearly 100% supplied by renewable energy sources. Ethiopia’s potential for renewable energy resources is immense, with an annual exploitable electric energy potential of 200TWh from hydropower, 4000TWh from wind energy, 7500TWh from solar energy [10] and 10GW from geothermal energy resources [11]. Despite the fact that these resources are not being used to generate electricity, the country is experiencing power shortages, with only 45% of the population having access to electricity [12]. Besides, Ethiopia’s current annual electricity consumption per capita is 133 kWh per year [13]. This is very low when compared to the average minimum level consumption per capita for a reasonable quality of life, which is 500 kWh per year [14].

Aside from the current challenges, power demand is expected to grow as the country targets a lower-middle-income economy by 2030 and sustaining growth to achieve medium and high middle-income country status by 2040 and 2050 respectively as detailed in “Homegrown Economic Reform Agenda (HERA)”. In addition, Ethiopia’s government presents an action plan to achieve universal electricity access across the country by 2030 [15] to aid the country’s economic transition. The action plan indicates that by 2030, power generation capacity would expand from the current 4.413 GW to 22.14 GW. In addition, the policy intends to increase electricity exports by 329% in 2030, from 0.35 GW to 1.5 GW. The action plan also intends to improve grid connectivity to 96% from the current grid connection of 65% and to limit stand-alone electrical access to 4%. Realizing Ethiopia’s development vision implies that the country needs adequate, reliable, affordable and environmentally sustainable electricity supply options. Achieving these required optimal generation capacity additions, which in turn considers diversification of power plants system. Finding optimal generation capacity additions based on least-cost planning is important in formulating supply options considering the high investment cost associated with it.

Energy models are increasingly used to support energy policymaking and investigate policy options and ambitious target settings [16] and offer in planning future investments towards a viable and sustainable power development [17]. It is a well-established practice to use energy models calibrated with high-quality data to support policymaking and energy planning activities in developed countries. However, this cannot be said for developing countries where financial availability and access to high-quality data are the major challenges. The Open Source Energy Modelling System (OSeMOSYS) was developed to fill a gap and was the result of a collaborative effort between several institutions [18]. In contrast to long-established energy system models, OSeMOSYS requires no upfront financial investment [19]. Previous studies have used OSeMOSYS to assess, among other things, for high penetration of renewable energy systems [20] and capacity planning in the context of uncertain climate policy cross-border electricity trade [21], and the balance of economic and environmental objectives [22]. As these studies demonstrate, OSeMOSYS applications play an important role in the energy planning process, emphasizing its societal significance.

Previous studies on energy issues in Ethiopia have looked at sustainable energy access [23], the potential for renewable energy resources [10], and energy projection [24, 25]. These studies however have limitations in accounting for the full level of national policy ambition in projecting demand and optimizing the supply-side energy mix to meet expected demand at the lowest possible cost. There has yet to be any research that has produced internally consistent national power system development pathways that account for the full range of national policy ambition. A study is thus required that provides policymakers with an optimal national power expansion pathway and an investment portfolio that specifically reflects the national development plan. It is therefore the objective of this study to apply OSeMOSY using the national development plan (HERA) as a scenario to provide the least-cost optimal energy supply option to meet national targets of becoming a lower-middle-income country by 2030, as well as a medium and higher middle-income country by 2040 and 2050 respectively.

The remainder of the paper is as follows: section 2 presents material and methods with emphasis on the description of OSeMOSYS, Reference Energy System (RES), basic model assumptions and data, model setup and the description of scenarios. The results are discussed in section 3 and section 4 concludes the discussion.

## 2. Materials and methods

The methodology applied in this study is centered on the plausible scenarios optimization representing the expansion of the power generation system to meet projected demand. The optimization was done in the Open Source energy Modelling System (OSeMOSYS) with a planning horizon from 2020 to 2050 adopted for this study.

### 2.1. Modelling tool

The Open Source energy Modelling Systems (OSeMOSYS/2017-11-08 version) was selected for this study. OSeMOSYS positions itself as a tool for educational purposes, capacity building, and fostering dialogues within and between institutions in the field of energy and integrated resource modelling. This is due to its structure, which is open, free to use, and easy to understand. For the same reasons, the tool is well suited to the analysis of energy resources-policy interfaces, such as the one addressed in this study. The objective function is to minimize the present value of expanding and operating the system components to meet exogenously defined energy demands. The ethos, structure, and aspects of the development of OSeMOSYS including model’s formulation, mathematical formulation, operation principle, implementation as well as a detailed description of the model inputs, parameters and outputs are described in [19]. The wide range of applications of OSeMOSYS as of 2018 including its dissemination, its use for sustainable policymaking and the link between modelling practice and the engagement with decision-makers are described in [18]. The updated model infrastructure is available on the GitHub repository of the tool [26] and the user manual is available on an open ReadTheDocs platform [27]. The original code of OSeMOSYS consists of several blocks of functionality, computing balances for costs, storage, capacity adequacy, energy balances and emissions.

### 2.2. Model setup and main assumptions

**Fig 1:**
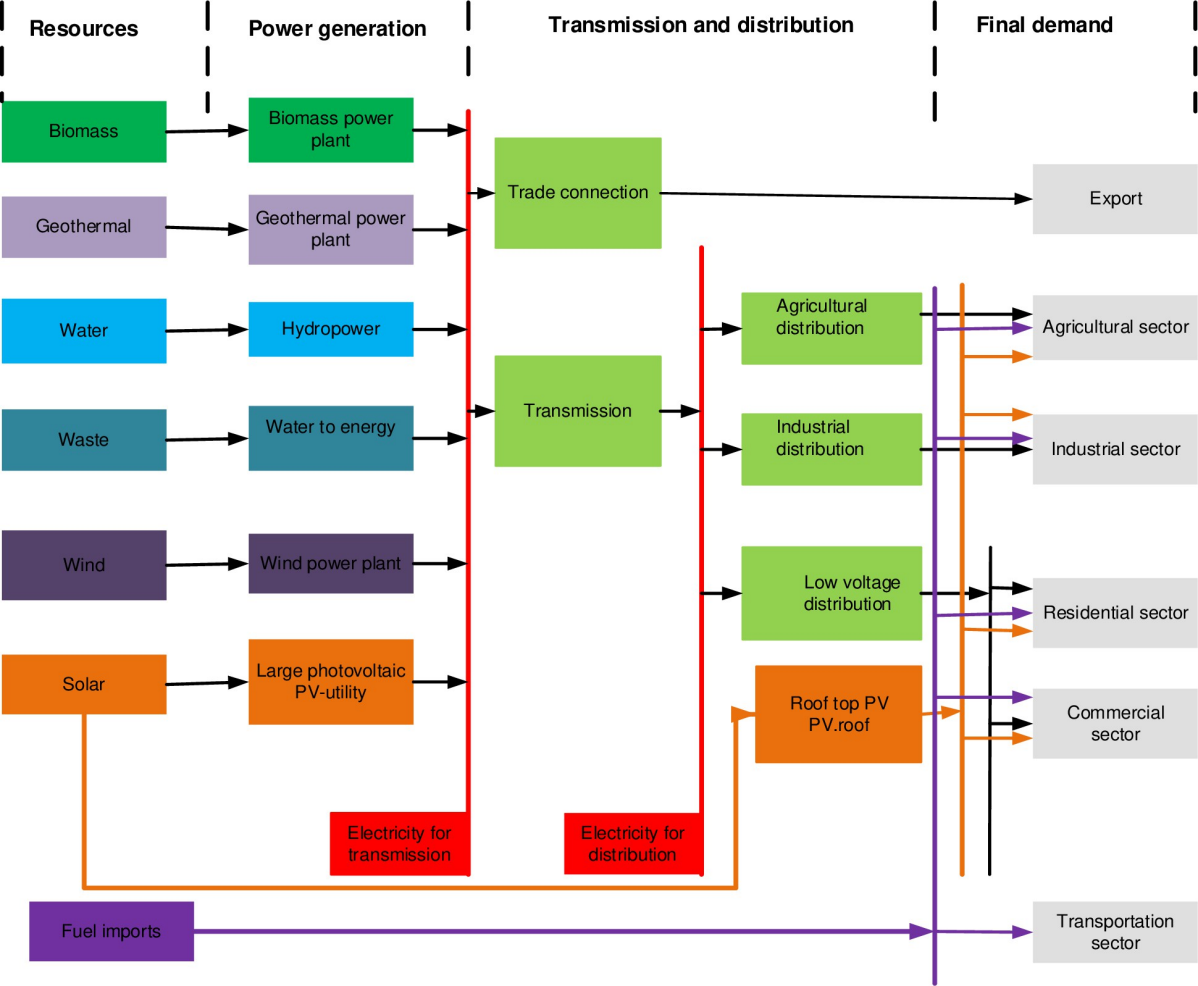
Simplified Reference Energy System (RES) of Ethiopia.

In OSeMOSYS modelling framework, the energy system is represented by four tiers including, primary energy resources supply, power generation technologies, transmission and distribution infrastructures and final demand sectors. The Reference Energy System (RES) has been created to represent a simplified and organized model structure of Ethiopia’s energy system (Fig 1). RES is a simplified and aggregated graphical representation of the real energy system that is being analyzed where each line represents energy flow and each box represents a technology. As indicated in RES, energy flows horizontally from the resource base on the far left, going through different transformation technologies, to reach final energy use on the far right and it is generally composed of three modules. Technologies can use and produce energy carriers, satisfy energy services and generate emissions as a by-product and represented by boxes in RES. Fuels include any energy vector, energy services or proxies entering or exiting technologies and is represented by lines in RES.

Being as an analytical tool representing the relationship between energy supply and demand with available energy conversion technologies from resource extraction to transformation, transmission/distribution of energy carriers and end use demand technologies, RES are affected by various factors. End-use demand is the driver of the system and is affected by several exogenous factors. The factors driving these demands differ across economic agents and sectors. In general, the residential sector consumes energy to satisfy certain needs and is affected by demography (population growth and urbanization), life style, weather and efficiency with which energy is used. Industries used energy as input of production and is affected by activity levels (output growth, industrial structure/shift in production, and efficiency with which energy is used. Activity levels within the sector and efficiency with which energy is used influence commercial and service sectors. Transport sector is influenced by transport activity level (the amount of transport), modes of transport used and efficiency of these modes.

Population growth, rapid urbanization and economic development are the main drivers for Ethiopia’s energy demand [28]. More specifically, a combination of total Growth Domestic Product (GDP) and electricity price are the drivers for the commercial sector and domestic demand are affected by demography and specific consumption [14] while the industrial GDP is the main driver for industrial energy demand. Further, the modelling outputs to supply the required demand at the end of the RES are a function of techno-economic parameters and constraints related to the functioning of the system. These parameters affecting the production capacity of the system includes technical parameters (input activity ratio, output activity ratio, residual capacity, availability factor, and operation life and capacity factor) and economic parameters (investment and operation costs). Fig 1 below presents a simplified RES for Ethiopia.

In developing a model, the RES has been introduced in the OSeMOSYS open-source energy-modelling framework, together with other parameters resulting in the OSeMOSYS-Ethiopian model. The model works by defining the least-cost mix of power technologies that should be deployed and operated to satisfy energy demand subjected to a set of technical and economic binding constraints. Techno-economic constraints (power plant capacities, capacity factors, efficiencies, lifetime, and costs) and environmental (CO2 emissions) parameters are assigned to the technologies. Final demand has been divided into five sectors and is being supplied by different energy chains based on the least cost option computed by the model for the time frame of 2020 to 2050.

As OSeMOSYS is demand-driven, energy demand is determined from scenario assumptions and interred into the model to determine energy supply and technology options. The study considers 2020 as the base year and the analysis covers 2020 to 2050. The discount rate has been set in the model as 10% based on the average discount rate used for Ethiopia [29, 30]. A straight method of depreciation is assumed and all monitory values are presented in US$. The national model is designed only for grid-connected electricity generation, transmission and distribution as Ethiopia plan to increase grid connection to 96% by 2030. Electricity loss for Ethiopia is 20% [31] and assumed accordingly. End-use electricity demands are combined into each respective sector demands. The load profile considered in the model includes three seasons (intermediate, summer and winter) and differentiate loads between day and night.

The existing capacity in the model is based on the Ethiopia power system expansion master plan study data set [15]. These data give information about the power plant concerning the installed capacity, when it was installed, and the operational life of the power plant. This information uses further to calculate the residual capacity of the existing power plants at the base year. Techno-economic data for electricity generation technologies have been derived from [32–34] and the related CO2 emissions factors have been derived from [35]. The cost of energy technologies is based on projected cost reductions for renewable energy technologies [34]. Fixed and variable costs are derived from [36]. Other constraints imposed in the OSeMOSYS concern the upper and lower bounds for endogenous variables are the energy resource potential and installed capacities limited based on national data [25].

### 2.3. Scenarios description

Considering the national vision of energy security and the importance of energy to support a national vision of achieving the middle-income country category in 2030 and beyond, two scenarios were considered.

#### 2.3.1. Business as Usual Scenario (BAUScen)

The overall assumption of this scenario considers energy consumption per capita continues consistent to the baseline year with implicit improvement related to urbanization and economic growth. The growth of the total demand is driven mainly by population and urbanization growth. Furthermore, it is assumed that the GDP growth rate of 11% per annum in the base year will decline gradually to 8.4% in 2030 and 6.5% in 2050 to consider economic fluctuation and anticipated decreasing tendency of the rate of GDP growth as the economy grows. The population growth was considered to grow from 112 million in a base year to 139.6 million by 2030 and 190.9 million by 2050 [37]. The rate of growth is considered to slow down from the current 2.43% average growth to 1.84% by 2030 and 1.3% by 2050. The current urban population of 20 million (~ 20%) will grow to 37 million by 2030 and 74.0 million by 2050. The current 4.63% rate of the urban population is also assumed to slow down to 3.86 and 3.02% by 2030 and 2050 respectively. The historical electricity access rate and per-capita electricity consumption rate are considered and will be assumed to be evolved in the future in the same way as they were in the past.

#### 2.3.2. Homegrown Economic Reform Agenda 2030 Scenario (HERA2030Scen)

The Ethiopian Government unveiled a "Homegrown Economic Reform Agenda" (HERA) aimed at unlocking the country’s development potentials designed to propel Ethiopia into becoming a lower-middle-income country by 2030. Based on the plan, the country graduates from a low-income country to lower-middle-income country status by 2030 as planned and continue to grow to a medium middle-income country by 2040 and a higher middle-income country by 2050. The key features of this scenario are the per capita electricity consumption of the country is assumed to match the current average consumption rates of lower-middle (760kWh/c), medium-middle (2064KEW/c) and higher middle (3496KWh/c) income country status by 2030, 2040 and 2050 respectively.

### 2.4. Demand projections

In the model, power demand is the driver of the model and are exogenously defined. As previously mentioned, in the model description expansion in capacity and investments are led by growing demands power. Two demand scenarios (BAUScen and RV2030Scen) are modelled based on assumptions of macro-level growth drivers. For BAUScen, demand projections are generated using correlations of historical data of consumptions with macro-level growth drivers. For HERA2030Scen, demand is generated based on the average per-capita consumption of electricity and macro-level growth drivers.

## 3. Results and discussion

Final electricity demands have been optimized to determine the optimal supply options for the Ethiopian electricity sector. This section presents OSeMOSYS modelling results calculated based on the least-cost energy supply options for electricity generation for the year 2020 to 2050. Two different energy supply options have been optimized in BAUScen and HERA2030Scen.

### 3.1. Model calibration

**Fig 2:**
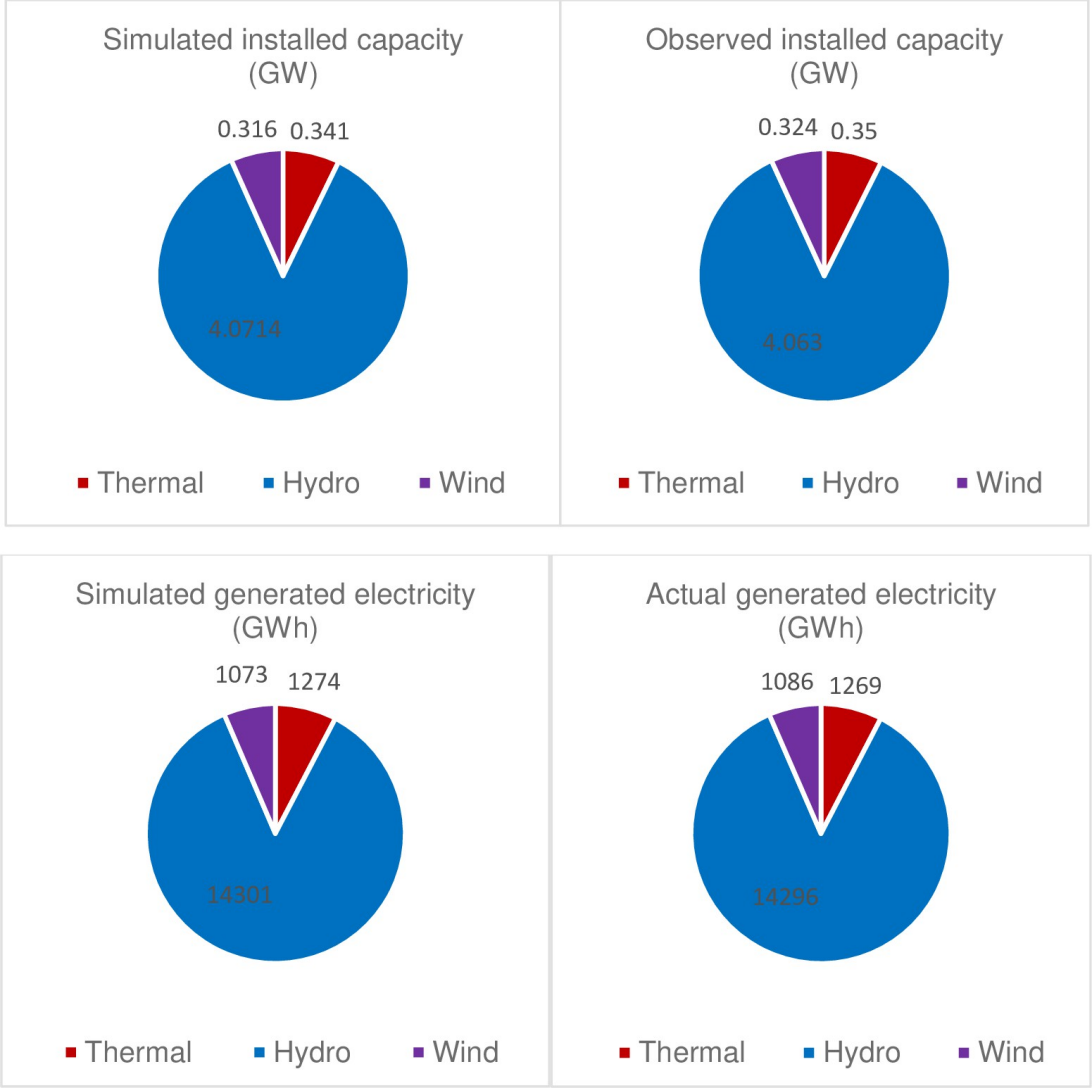
Model calibration for the base year.

For the model calibration, the model results were compared against the actual data for the base year (2020). As indicated in Fig 2, it is clear that the model captures the historical installed capacity and the power mix.

### 3.2. Installed capacity

The model results show that to maintain the current electricity consumption pattern (BAUScen), Ethiopia requires an increase in installed capacity from the base year of 4.213GW to 11.24GW, 12.06GW and 13.68GW by 2030, 2040 and 2050 respectively. For HERA2030Scen, the power capacity needs to grow to 31.22GW, 112.45GW and 334.27GW by 2030, 2040 and 2050 respectively. The least-cost optimal results show HERA2030Scen has the highest total capacity additions at 178% as compared to BAUScen by 2030. This indicates that the BAUScen power expansion trend does not fulfil the homegrown target of achieving lower-middle-income status by 2030. The expansion of power plant capacity in the Ethiopian power system based on designed scenarios is illustrated in Fig 2 [subplot a]. Our estimate of 31.22GW is 9.08GW more than the national power development plan of 22.14GW by 2030 [15]. This disparity results from the national projection of power expansion failing to account for the current lower-middle-income per-capita consumption target.

According to BAUScen, hydropower will continue to be the primary source of national electricity generation, accounting for 89% and 73% of total installed capacity by 2030 and 2050, respectively. Wind power is the second contributor with 9.9% and 26.8% by 2030 and 2050 respectively. Together, Hydropower and wind power are the most optimum contributing with 98.9% and 99.8% of the total installed capacity by 2030 and 2050. For the HERA2030Scen, the structure of power generation capacity is expected to change over time, mainly due to the exhaustion of the available hydropower potential. The optimum installed capacity mix shows that by 2030; hydropower, wind, and geothermal shares 57%, 26% and 16% and others (biomass and waste) 1% of the total installed capacity respectively. The least-cost generation mix differs from the national plan anticipates a capacity expansion mix of hydro (75.9%), wind (9%), geothermal (3.8%), and Others (Biomass and waste (3.2%) [15]. The disparity in power generation capacity mix is primarily explained by the fact that the national plan didn’t apply a cost optimization model. By 2050 the installed capacity shifts to 48% wind, 34% solar, 13% hydro and 2.9% geothermal. The enormous required increase in power production by 2050 is associated with the assumption that Ethiopia moves progressively from a lower-middle-income country by 2030, to a medium middle-income country by 2040 and upper middle income by 2050.

### 3.3. Least cost electricity generation mix

**Fig 3:**
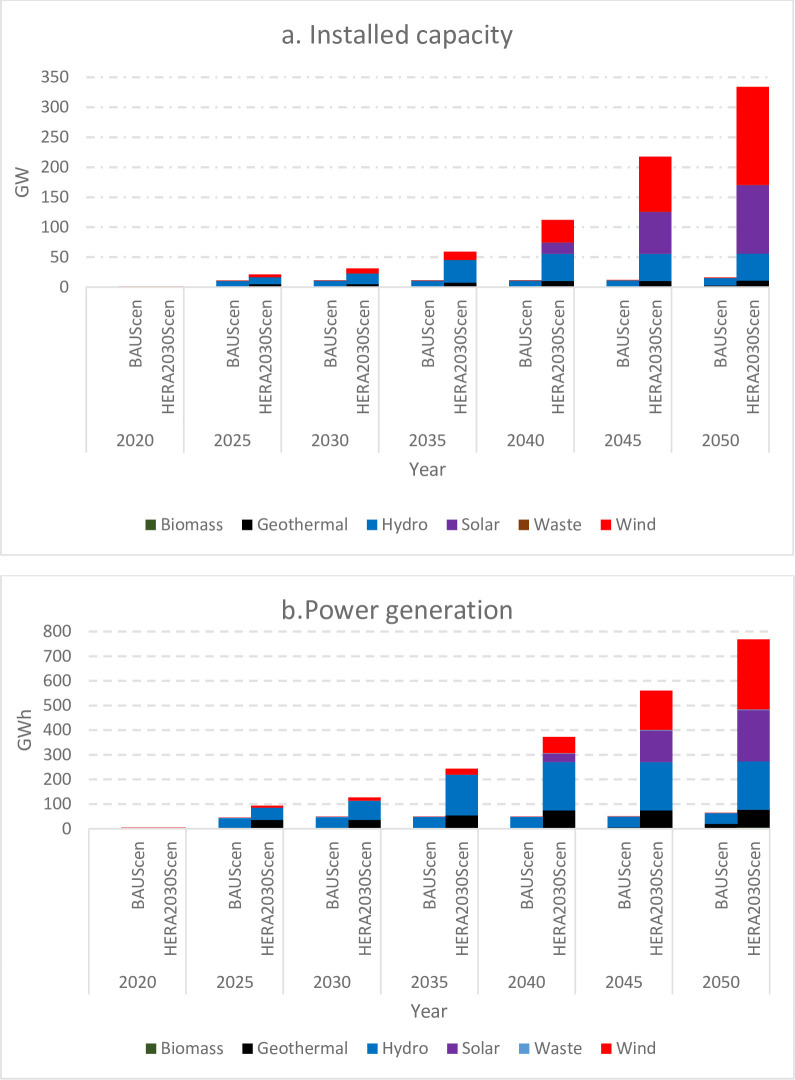
Least cost total installed capacity [a] and electricity generation [b] for the study period (2020–2050).

Generated electricity based on the designed scenarios is show in Fig 3 [sub-plot b]. Generated electricity based on the BAU scenario reaches 49.47TWh and 65.73TWh by 2030 and 2050 respectively. These products will increase by 328% in 2030 and 540% in 2050 compared to base year generation. Electricity will mainly produce by technology running on hydropower. Alone, hydropower generates 35.23 TWh (71%) in 2030. Though hydropower is technically a clean energy source, harnessing it for large amounts of power has environmental and social consequences. Though, many natural sites in Ethiopia are well suited to the construction of storage reservoirs with minimal environmental and social impact [38], proper planning and careful system design are required for long-term hydropower development to manage the challenge.

Under the HERA2030Scen, the total electricity generation peaks to 127.80TWh and 768.88TWh by 2030 and 2050 respectively. Compared to BAUScen, this is an increase of 158% and 1454% by 2030 and 2050 respectively. The generation mix has undergone significant change with increasing electricity demand where hydropower share reduces to 57% in 2030 and 13% in 2050. Besides, to replace hydropower, solar energy will penetrate at the highest rate towards 2050. In 2039 solar power technology starts to make a significant share of the electricity generation of 33.94 TWh, and increases to 208.08TWh by the end of the presented period time (2050). As a result, national energy generation gradually shifts to solar technologies towards 2050 and solar and wind technologies are the most used technologies towards 2050.

From the results, it is indicated that with the hydropower and geothermal potential reaching their maximum exploitable limits, solar and wind technologies are expected to contribute Ethiopia’s ambitious electrification paths. However, the stochastic nature of solar and wind energy sources are usually threatens the stability of the electricity grid. Specifically, solar and wind are the intermittent source of renewable energy sources that would not able to adjust supply in the case of a sudden change in the demand. This makes the penetration of solar and wind energy technologies the most challenging issue in power systems.

Despite these challenges, rising electricity demand and awareness of the need to keep global warming below 1.5°C in order to meet internationally agreed climate change targets necessitate a major transformation of energy systems that dramatically increases the integration of intermittent renewable energy sources such as wind [39–42] and solar [43–46]. The dynamic thermal rating (DTR) and energy storage systems [47–49] as well as demand response to actively manage load profiles [50–55] are pioneering approaches to enhance the reliability of the power network and increase the penetration of such variable renewable energy sources into power systems.

In this regard, outstanding studies conducted on the impact of DTR on wind energy integration [1, 49, 56, 57] addresses its potential benefits of increasing the penetration of wind energy resources to power system generation grid. DTR also provides a higher current-carrying capacity for transmission lines and thus can mitigate system congestion and reduce generation dispatching in the cases when congestion is caused by the transmission thermal limit [51]. These studies demonstrated that the implementation of a DTR system could relieve congestion on transmission lines and by doing so, increase the penetration of solar and wind energy technologies.

Other studies also articulate the ability of DTR to provide an adequate and secure power supply while taking into account transmission system constraints [58], the reliability impacts of the DTR system on power grids while taking into account the wireless communication network [59], and the integration of DTR and operational tripping scheme (OTS) to avoid unnecessary generation tripping due to conservative line ration [60]. From these studies, it has been proved that the deployment of DTR can help to enhance power system reliability and renewable energy integration. In addition, by coordinating DTR with other smart grid technologies, the potential benefit may be more dramatic. Further, demand reduction in combination with dynamic thermal line rating systems can optimize network reliability and ageing, while demand losses are minimized [54], which refers to the reduction or shifting the power utilization during top periods in response to time-sensitive rates or different types of monetary motivating force.

With the increasing role of variable power generation from solar and wind, battery storage systems are other technology to maintain the balance to enhance renewable energy penetration. Grid level batteries can store energy when there is excess generation from wind and solar and discharge it to meet variable peak demand. Optimized battery energy storage system can minimize the power curtailment, network ageing, and increase reliability [40], reduces the renewable energy systems curtailment considering power flow constraints [61] and increase the reliability of generation by allowing the direct load reduction due to equipment shutdown as well as load redistribution during the day [39]. Employing a DTR system in a power network is therefore the potential to improve network capacity, increase the utilization of existing assets of solar and wind energy sources. This suggests that, despite Ethiopia’s existing hydropower reliance, solar and wind energy technologies should be considered feasible investment prospects during the planning phase if the country is to meet its ambitious electrification goals.

Contrary to our findings, previous studies that projected power demand to be111.4TWh by 2037 [24] and 119.7TWh by 2045 [25] underestimates the power demand required to achieve national target. The projected power demand from these studies translated to the per-capita power consumption of 703KWh/c and 669 KWh/c respectively, which is very low, compared to the expected average per-capita consumption of 1259KWh/c in 2037 and 2780 KWh/c by 2045 based on countries economic transition to middle-income countries. This indicates that demand is thus underestimated. As the previous analysis considers only access rate without objectively defining the average per-capita consumption target as per the middle-income country status, thus could have an impact on the power expansion capacity to meet the targeted national demand. Moreover, the previous study lacks to optimize the supply-side energy mix to meet the projected demand. Besides, Ethiopia has sufficient potential renewable energy sources to cover all national demands including power export as planned in HERA target. Compared to the national power generation potential of more than 11, 700TWh from renewable energy resources [10], the projected energy demand of 768.88TWh is by far lower than the national generation potential. National electricity demand to achieve middle-income country status will be met with the development of 100% clean energy sources.

### 3.4. Financial requirements

The capital investment cost required for the entire period of study is based on OSeMOSYS least-cost modelling results. Achieving national target lower-middle-income status worth an aggregate expenditure of 70 billion US$ from 2021 to 2030, a 7.74 billion US$ annually and translated to a total per-capita expenditure of 494 US$. This is 151% higher than the BAUScen. To achieve upper-middle-income electricity usage rates in 2050 (from 2031 to 2050 inclusive), a cumulative cost of 750 billion US$ will be spent, amounts to an average of 37.66 billion US$ per year and translated to a total per-capita expenditure of 3945 US$. There is a discrepancy between our estimate and investment cost estimated by MoEI for HERA by 2030 for universal electrification to be 23.58 US $ [15] which is low compared to our estimate of 70 US $. The discrepancy is due to the fact that the national estimate didn’t project the per-capita consumption to the current middle-income country level.

**Fig 4:**
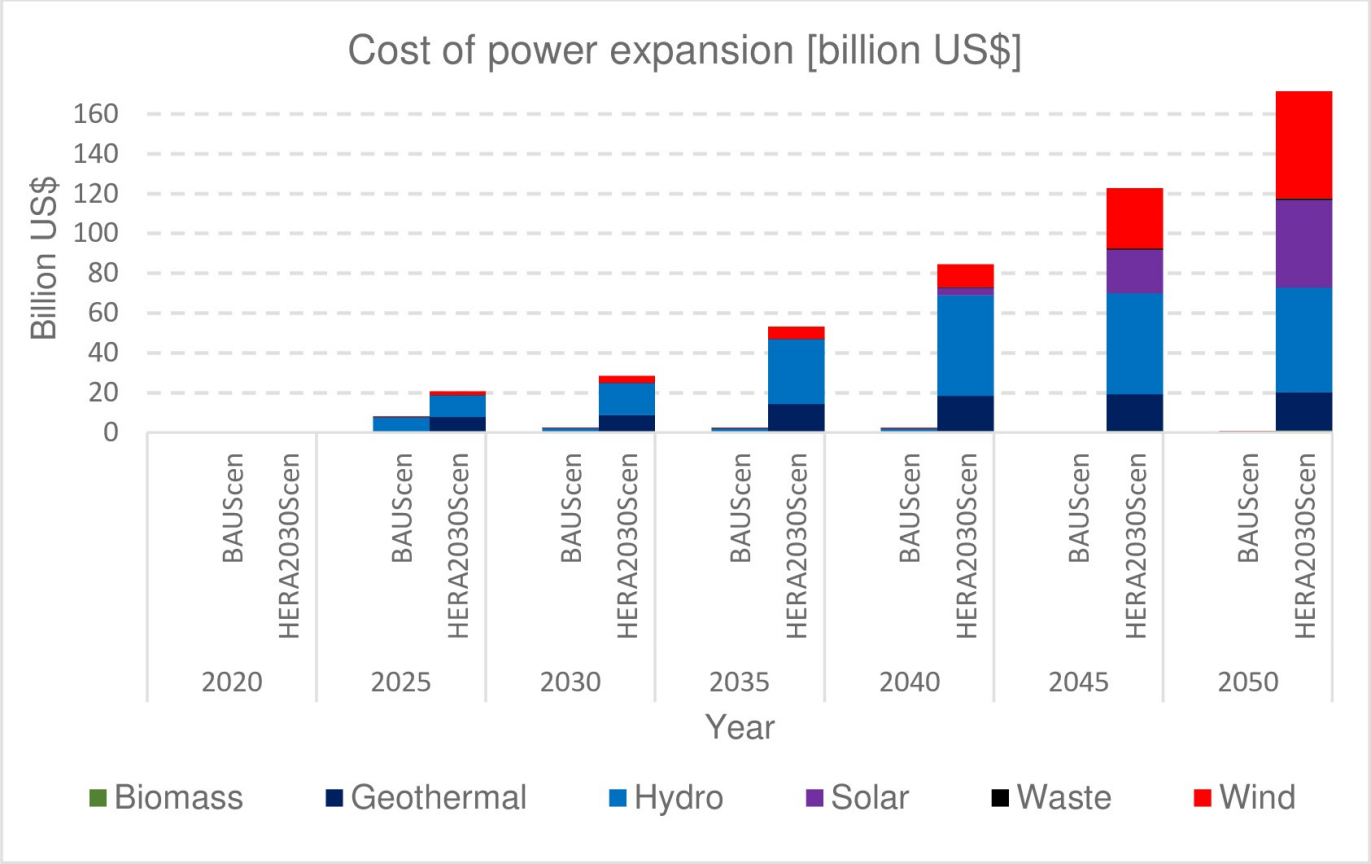
Investment cost of power capacity expansion.

Compared to International Energy Agencies (IEA) cost prediction of renewable energy technologies, which does not include the learning curve, the total cost of electricity production to achieve the HERA2030Scen demand target is 6% and 13% lower by 2030 and 2050 respectively. These trends are likely to continue over time because of the process of “technological learning” [62]. Understanding how the costs of renewable power supply technologies change over time is of key importance for decision-makers concerned with power development. The cumulative sharing of capital investment for the period of 2020 to 2050 at a five-year interval is as shown in Fig 4.

As our analysis and a previous study [63] showed, Ethiopia has significant renewable energy resources potential to meet all national demands. Thus, Ethiopia can meet ambitious national electrification and long-term social and economic goals through greater reliance on indigenous renewable energy sources. However, the most daunting challenge the country face is economic energy scarcity. Ethiopia is a developing country in the early stages of industrialization, with a potential issue in ensuring energy security due to a lack of financial resources. Public funding alone may not be sufficient to support such financial requirements for energy infrastructure and service provision. Ethiopia plans to increase private participation in the power sector from the current zero levels to 4GW by 2030. However, compared to huge financial requirements to achieve the national vision set by HERA2030, political decisions are needed to promote and attract large investment and capacity building activities.

## 4. Conclusion and policy implication

The study presented a modelling approach on the renewable-based energy supply options for electricity generation in Ethiopia. The modelling approach emphasized optimal results based on the least cost assumed in OSeMOSYS. Ethiopia has enough renewable energy resource potential to meet the national plan’s target of increasing grid connections to 96% by 2030. The outcome demonstrates how various power generation technologies and energy supply mix can be chosen to meet projected national power demand at the lowest possible cost. Major investments in power infrastructure will be needed in the coming decades due to the increasing electricity demand. The cost of achieving the national target of 100% access rate and middle-income country status from the current low access rate and consumption rate involves a substantial increase in total investment cost (151% increase compared to BAUScen). Economic energy scarcity is, therefore, a daunting challenge for a country to meet the national target of energy security. To overcome this issue, political decisions are needed to promote and attract large investment and capacity building activities.

The insights gained from this study could help decision-makers gain a comprehensive understanding of the options for optimum development path for renewable energy resources. With a better understanding of the power sector evolution, policymakers responsible for long-term expansion planning will make better-informed decisions to update current national plans, support policy and future investment decisions in the energy sector. The study supports Ethiopia’s vision of transformation from traditional to modern energy sources and drives a country to be a renewable energy hub in the region, a well-intentioned policy path to pursue. It is also consistent with carbon-neutral economic growth ambition in its green growth strategic plan [64] and allows Ethiopia to its clean and sustainable energy development goal. There are several limitations to the analysis in this paper, some of which can be addressed in further work. Despite sufficient renewable energy resources for power generation, Ethiopia currently relies on imported fuels for the transportation sector and its emission scenario will need further consideration. Moreover, potential improvement in technology efficiency will need to be further evaluated and implemented in the model.

## Supporting information

S1 Data (XLSX)
